# MCP-1/CCR2 signaling-mediated astrocytosis is accelerated in a transgenic mouse model of SOD1-mutated familial ALS

**DOI:** 10.1186/2051-5960-1-21

**Published:** 2013-06-04

**Authors:** Motoko Kawaguchi-Niida, Tomoko Yamamoto, Yoichiro Kato, Yuri Inose, Noriyuki Shibata

**Affiliations:** 1 Department of Pathology, Tokyo Women’s Medical University, 8-1 Kawada-cho, Shinjuku-ku, Tokyo 162-8666, Japan

**Keywords:** Amyotrophic lateral sclerosis, Astrocyte, CCR2, MCP-1, Motor neuron, SOD1

## Abstract

**Background:**

Emerging evidence suggests that innate immunity and increased oxidative stress contribute to pathomechanisms in amyotrophic lateral sclerosis (ALS). The aim of the present study was to verify the involvement of monocyte chemoattractant protein-1 (MCP-1) and its specific CC chemokine receptor 2 (CCR2) in the disease progression of ALS. We here demonstrate the expression state of MCP-1 and CCR2 in lumbar spinal cords of mice overexpressing a transgene for G93A mutant human superoxide dismutase 1 (SOD1) (ALS mice) as a mouse model of ALS as well as the involvement of MCP-1/CCR2-mediated signaling in behavior of cultured astrocytes derived from those mice.

**Results:**

Quantitative polymerase chain reaction analysis revealed that MCP-1 and CCR2 mRNA levels were significantly higher in ALS mice than those in nontransgenic littermates (control mice) at the presymptomatic stage. Immunoblot analysis disclosed a significantly higher CCR2/β-actin optical density ratio in the postsymptomatic ALS mouse group than those in the age-matched control mouse group. Immunohistochemically, MCP-1 determinants were mainly localized in motor neurons, while CCR2 determinants were exclusively localized in reactive astrocytes. Primary cultures of astrocytes derived from ALS mice showed a significant increase in proliferation activity under recombinant murine MCP-1 stimuli as compared to those from control mice.

**Conclusions:**

Our results provide in vivo and in vitro evidence that MCP-1 stimulates astrocytes via CCR2 to induce astrocytosis in ALS with SOD1 gene mutation. Thus, it is likely that MCP-1/CCR2-mediated sigaling is involved in the disease progression of ALS.

## Background

Amyotrophic lateral sclerosis (ALS) is a late onset neurodegenerative disease characterized by a progressive and selective loss of motor neurons in the motor cortex, brain stem motor nuclei, and spinal cord ventral horns [1]. Patients affected with ALS develop progressive muscle weakness associated with neurogenic amyotrophy, and they will die of respiratory failure within 3–5 years unless undergoing artificial ventilation [2]. Approximately 10% of the ALS patients are familial. About 20% of the familial ALS patients are associated with mutations in the gene for superoxide dismutase 1 (SOD1) [1]. Mice carrying a transgene for the mutant human SOD1 gene demonstrate clinicopathological features resembling human ALS [3]. Thus, mutant human SOD1 transgenic mice have been used in a large number of studies on ALS as an outstanding animal model of ALS.

Although the complete pathomechanism of ALS has not yet been understood, several studies have obtained evidence that inflammatory processes, including increased levels of proinflammatory cytokines and proliferation and activation of glial cells in the main lesions, are involved in the disease progression [4]. Actually, our previous report showed increased levels of activated form of p38 mitogen-activated protein kinase (MAPK) and reduced levels of inhibitor of kappa B-alpha (IκBα) in G93A mutant SOD1 transgenic mice as well as a beneficial effect of pioglitazone, an antiinflammatory agent of the thiazolidinedione group and an artificial agonist of peroxisome proliferator-activated receptor gamma, on survival of motor neurons and suppression of glial activation through inhibition of p38 MAPK activation and upregulation of IκBα expression [5].

As reviewed by Conductier et al., several investigations have demonstrated implications for monocyte chemoattractant protein-1 (MCP-1), a synonym of CC chemokine ligand 2 (CCL2), in neurological disorders [6]. MCP-1, an 8 kDa secretory protein, is released from certain cells to exert a potent proinflammatory effect on its target cells by binding to the specific receptor CCR2 [7]. MCP-1/CCR2-mediated signaling drives the downstream phosphatidylinositol-3 kinase/Akt and MAPK pathways [8-10]. It is known that MCP-1 induces chemotaxis of macrophages and microglia, leading to pathological microgliosis and inflammatory activation in the lesions [11]. This is supported by a number of studies showing that MCP-1 knockout mice are resistant to stroke and autoimmune encephalomyelitis [12,13].

Recent studies have suggested implications for MCP-1 in ALS. Increased levels of MCP-1 in serum or cerebrospinal fluid of sporadic and familial ALS patients [14-18] or spinal cord tissue samples from mutant SOD1 transgenic mice [19,20] have been reported. On the other hand, it is of interest that CCR2 expression levels on the cell surface of circulating monocytes in sporadic ALS patients were very low [21,22]. However, the role of CCR2 in a mouse model of ALS remains to be determined. To address this issue, we evaluated the expression state of CCR2 as well as MCP-1 in the spinal cord of mutant human SOD1 transgenic mice, by quantitative and morphological approaches using a reverse transcription-quantitative polymerase chain reaction (RT-qPCR), immunohistochemistry, and immunoblotting techniques. We also evaluated in vitro effects of MCP-1 using primary cultures of astrocytes derived from the transgenic mice and nontransgenic littermates.

## Results

### MCP-1 and CCR2 mRNA levels are changed in the spinal cord of ALS mice

**Figure 1:**
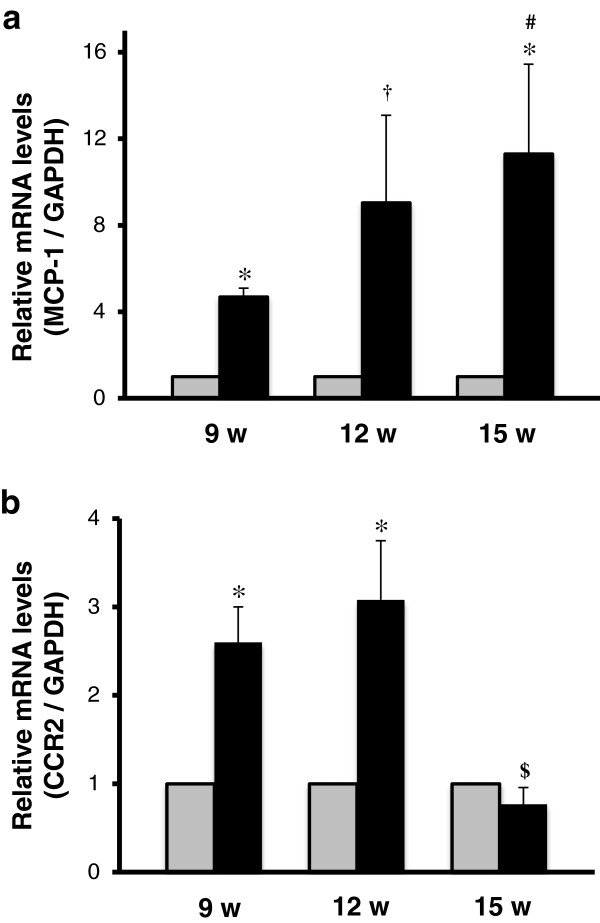
**RT-qPCR analysis for MCP-1 and CCR2 mRNA in the spinal cord of mice.** MCP-1 (**a**) and CCR2 (**b**) mRNA levels normalized with GAPDH mRNA levels are compared between SJL (gray columns) and G1H+/− (black columns) mice sacrificed at presymptomatic (9 w), onset (12 w), and postsymptomatic (15 w) stages (n = 6 in each group). Two-way ANOVA provides P < 0.05. Posthoc Bonferroni correction provides ^#^P < 0.05 and ^$^P < 0.01 as compared to the presymptomatic and onset G1H+/− groups and *P < 0.01 and ^†^P < 0.001 as compared to the age-matched SJL groups.

Using RT-qPCR techniques, expression levels of MCP-1 and CCR2 mRNA in lumbar spinal cords from G1H+/− (ALS mice) and SJL (control mice) mice were quantitatively compared between the presymptomatic (9-weeks-old mice), onset (12-weeks-old mice), and postsymptomatic (15-weeks-old mice) groups. MCP-1 mRNA analysis revealed clear results (Figure 1a). In all of these stages, MCP-1 mRNA levels were significantly higher in the G1H+/− groups than those in the age-matched SJL groups and age-dependently increased in the G1H+/− groups but not the SJL groups. On the other hand, CCR2 mRNA analysis revealed complicated results (Figure 1b). CCR2 mRNA levels were significantly higher in the presymptomatic and onset G1H+/− groups than those in the age-matched SJL groups, whereas there was no significant difference in the levels between the postsymptomatic G1H+/− group and the age-dependent SJL group. In G1H+/− mice, CCR2 mRNA levels tended to be higher in the onset group than that in the presymptomatic group, and were significantly lower in the postsymptomatic group than in the other groups. By contrast, SJL mice showed constant CCR2 mRNA levels among the three stage groups.

### MCP-1 protein is mainly expressed in spinal cord motor neurons of ALS mice

**Figure 2:**
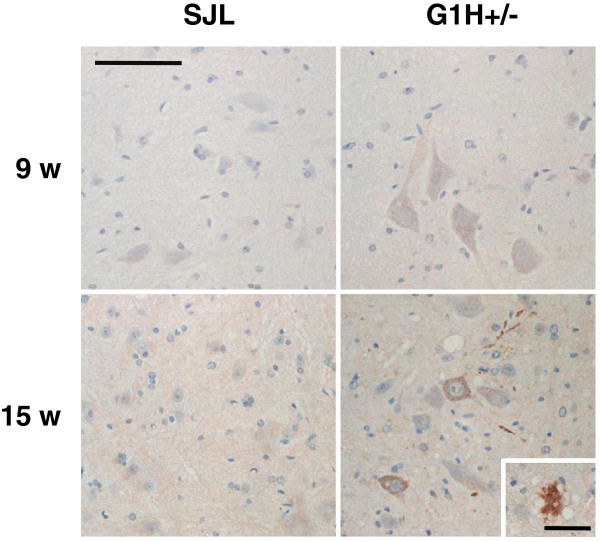
**Immunohistochemical observations of MCP-1 protein in the spinal cord of SJL and G1H+/− mice sacrificed at presymptomatic (9 w) and postsymptomatic (15 w) stages (n = 3 in each group).** Inset indicates a vacuolated neuron. Immunoreaction product deposits are visualized by the avidin-biotin-immunoperoxidase complex method using 3,3’-diaminomenzidine tetrahydrochloride and hematoxylin as the chromogen and counterstain, respectively, by light microscopy. Scale bars indicate 100 μm (panels) and 50 μm (inset).

MCP-1 immunohistochemistry made a striking contrast between G1H+/− and SJL mice (Figure 2). While MCP-1 immunoreactivity was distinct in pre- and postsymptomatic G1H+/− mice, it was only very weak or not at all in the age-matched SJL mice. In G1H+/− mice, immunoreactivity was mainly detectable in the cytoplasm of motor neurons, was more intense in the postsymptomatic group, and was prominent in vacuolated neurons, in particular, but was very weak in glial cells.

### CCR2 protein is mainly expressed in spinal cord reactive astrocytes of ALS mice

**Figure 3:**
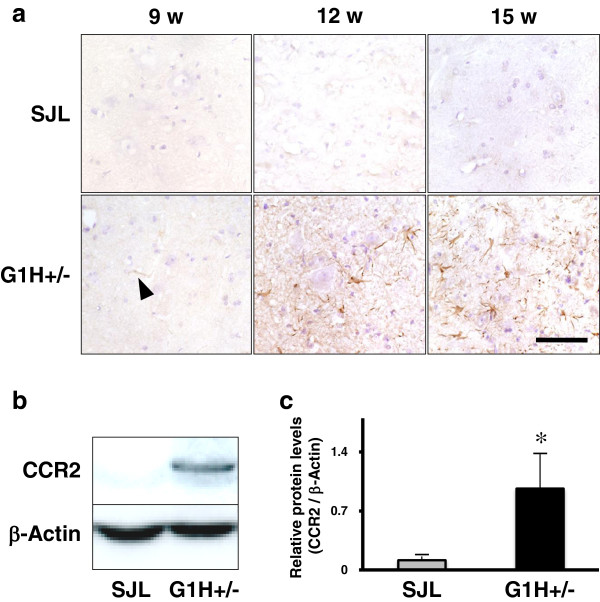
**Immunohistochemical (a), immunoblot (b) and densitometric (c) analyses for CCR2 protein in the spinal cord of SJL and G1H+/− mice sacrificed at presymptomatic (9 w), onset (12 w) and postsymptopatic (15 w) stages.** Immunoreaction product deposits are visualized by the avidin-biotin-immunoperoxidase complex method using 3,3’-diaminomenzidine tetrahydrochloride and hematoxylin as the chromogen and counterstain, respectively, by light microscopy. Scale bar indicates 100 μm (**a**). Electrophoretic mobility (**b**) and optical density (**c**) are compared between the postsymptomatic SJL and G1H+/− groups (n = 5 in each group). Two-way ANOVA provides P < 0.05. Posthoc Bonferroni correction provides *P < 0.05 as compared to the SJL group.

**Figure 4:**
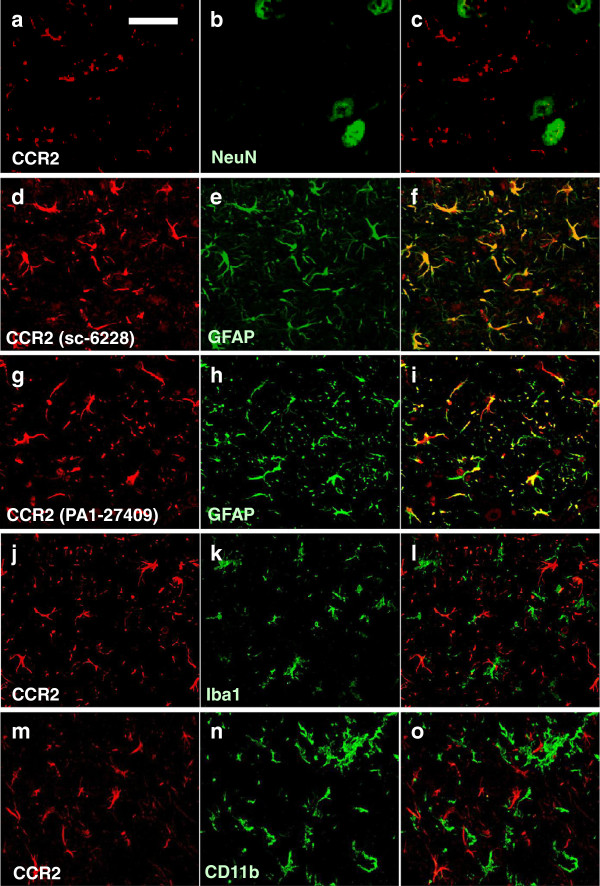
**Immunohistochemical observations of CCR2 protein in spinal cord ventral horns from G1H+/− mice sacrified at onset stage (12 w).** Localization of CCR2 immunoreactivity is verified by comparison with that of immunoreactivities for NeuN-immunoreactive **(b)** neurons, GFAP-immunoreactive **(e, h)** astrocytes, and Iba1-immunoreactive **(k)** and CD11b-immunoreactive **(n)** microglia. CCR2 immunoreactivity is detected with the two different antibodies sc-6228 **(a, d, j, m)** and PA1-27409 (g), respectively. Panels **(c, f, i, l, o)** indicate merged images in two other panels of each line. Immunoreactive signals are detected by the double-labeled immunofluorescence method using secondary antibodies conjugated with Cy3 (red) or FITC (green). Scale bar indicates 50 μm **(a-o)**.

**Figure 5:**
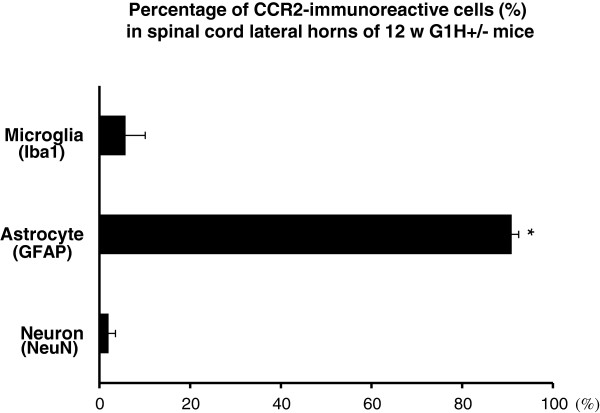
**The percentage of CCR2-immunoreactive cells in neurons, astrocytes and microglia.** Data obtained by the double-labeled immunofluorescence method are compared by two-way ANOVA (P < 0.01) and posthoc Bonferroni correction (*P < 0.01 as compared to the neuronal and microglial groups).

CCR2 immunoreactivity also showed distinct alterations between SJL and G1H+/− mice (Figure 3a). The immunoreactivity was only very weak in young to old SJL mice and presymptomatic G1H+/− mice. By contrast, it was highly intense in onset and postsymptomatic G1H+/− mice, and was particularly prominent in glial cells, but was undetectable in neurons. To identify CCR2-immunoreactive cells, we performed double-labeled immunofluorescence staining of sections from G1H+/− mice at onset stage. CCR2 immunoreactivity was detected in almost all GFAP-immunoreactive astrocytes (Figure 4d-f; g-i), whereas it was detected in only a few NeuN-immunoreactive neurons (Figure 4a-c) and Iba-1 or CD11b-immunoreactive microglia (Figure 4j-l; m-o). There was no significant difference in staining patterns between the two different anti-CCR2 antibodies. These results were confirmed by quantitative image analysis; the great majority of CCR2-immunoreactive cells in spinal cord ventral horns were astrocytes but not neurons or microglia (Figure 5).

### CCR2 protein levels are increased in the spinal cord of ALS mice

Expression levels of CCR2 protein in lumbar spinal cords were quantitatively compared between the postsymptomatic SJL and G1H+/− groups. Immunoblot analysis disclosed CCR2-immunoreactive signals, prominent in the G1H+/− group, at a mobility of 42 kDa (Figure 3b). Densitometric analysis revealed that immunoreactive signals for CCR2 normalized with those for β-actin were significantly higher in the G1H+/− group than in the age-matched SJL group (Figure 3c).

### rmMCP-1 induces proliferation of cultured astrocytes derived from ALS mice via CCR2

**Figure 6:**
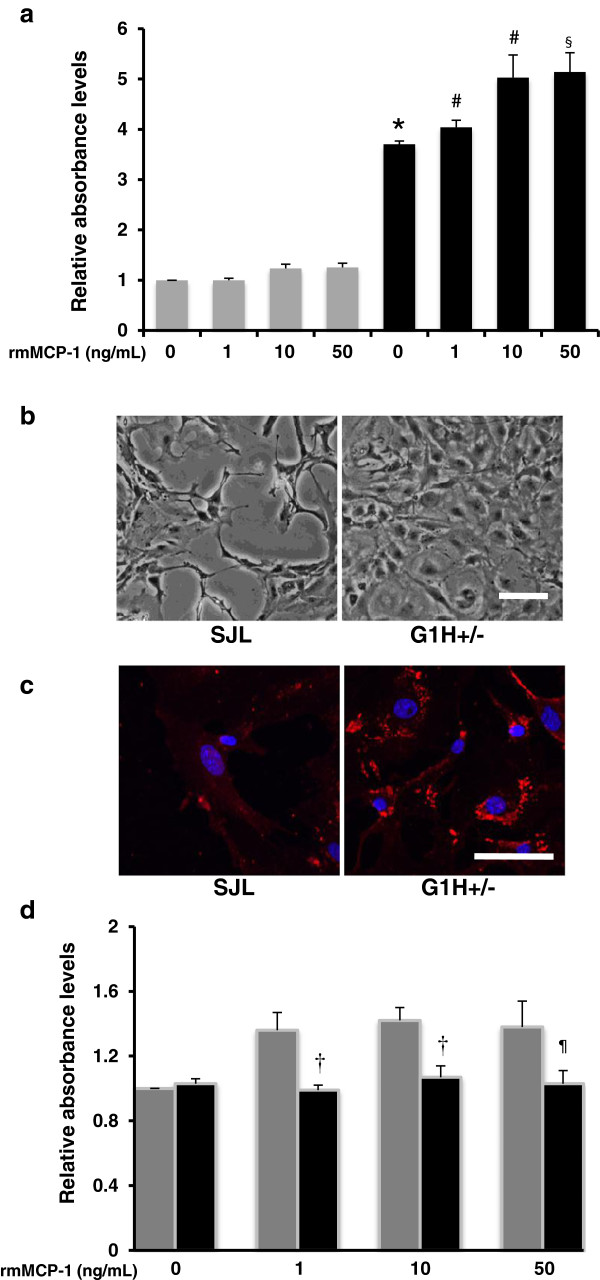
**Effects of MCP-1 on proliferation activity of astrocytes derived from SJL and G1H+/− mice.** Cultured astrocytes derived from SJL (gray columns) and G1H+/− (black columns) mice are stimulated with recombinant murine MCP-1 (rmMCP-1) at concentrations of 0, 1, 10 and 50 ng/mL for 48 h, and the proliferation activity determined by a CCK8 kit is compared **(a)**. The G1H+/− astrocytes are also stimulated with 10 ng/mL rmMCP-1 in the presence (black columns) or absence (gray columns) of treatment with 10 μM CCR2 antagonist, and the proliferation activity is compared **(d)**. Two-way ANOVA provides P < 0.05 **(a, d)**. Posthoc Bonferroni correction provides *P < 0.001 as compared to the MCP-1-unstimulated SJL cell group, ^#^P < 0.05 and ^§^P < 0.01 as compared to the MCP-1-unstimulated G1H+/− group, and ^¶^P < 0.05 and ^†^P < 0.01 as compared to the CCR2 antagonist-untreated, rmMCP-1 concentration-matched G1H+/− groups. Morphological changes of cultured astrocytes stimulated with 10 ng/mL rmMCP-1 are compared between the SJL and G1H+/− groups by phase-contrast images (b) and CCR2 immunocytochemistry detected by the immunofluorescence method using a secondary antibody conjugated with Cy3 (red) and DAPI (blue) as a nuclear marker **(c)**. Scale bars indicate 50 μm **(b, c)**.

Using primary cultures, we compared effects of MCP-1 on the proliferative activity of primary astrocytes derived from SJL and G1H+/− mice, as determined by a CCK-8 kit. In the absence of rmMCP-1, the basal levels of proliferation activity of astrocytes were significantly increased in the G1H+/− group as compared to the SJL group. In the presence of rmMCP-1, the levels exhibited a dose-dependent increase in the G1H+/− groups but not the SJL groups (Figure 6a). Phase-contrast images verified an increased density of astrocytes derived from G1H+/− mice as compared to those from SJL mice (Figure 6b). CCR2 immunoreactivity was intense and localized in the cytoplasm of astrocytes derived from G1H+/− mice, whereas it was only weak in astrocytes derived from SJL mice (Figure 6c). To determine whether the MCP-1-driven proliferation of astrocytes derived from G1H+/− mice may be mediated by the specific receptor CCR2 stimulation, we evaluated the influence of the CCR2 antagonist on the proliferation activity. As a consequence, the levels were significantly reduced in the antagonist-treated G1H+/− groups as compared to the rmMCP-1 concentration-matched, antagonist-untreated G1H+/− groups (Figure 6d).

## Discussion

### Morphological and quantitative evaluations for MCP-1 in SOD1-mutated mice

It is known that MCP-1 is upregulated by oxidative stress and inflammatory stimuli associated with several pathological conditions including inflammatory and autoimmune diseases and injuries [23,24]. Expression patterns of MCP-1 in the central nervous system (CNS) of postnatal mammalians have been well described. Under physiological conditions, MCP-1 is constitutively expressed in various types of cells, such as neurons, astrocytes, microglia, and endothelial cells at a minimal level. By contrast, it is highly induced in these cells or peripheral blood-derived monocytes, T cells, or natural killer cells under pathological conditions such as traumatic injury, excitotoxicity, ischemia, inflammation, and neurodegeneration [25-31].

As reviewed by McCombe and Henderson, emerging evidence suggests the involvement of proinflammatory mechanisms in ALS. Recent studies have demonstrated increased expression levels of proinflammatory cytokines and chemokines in activated microglia and reactive astrocytes in human ALS and its transgenic mouse models [32,33]. Several studies indicated increased expression levels of MCP-1 in the spinal cord of sporadic ALS patients and SOD1-mutated mice [20]. Other investigators demonstrated the correlation between the cerebrospinal fluid MCP-1 levels and the disease progression and severity of ALS [33,34].

In the present study, immunohistochemical analysis revealed that MCP-1 determinants were mainly localized in the cytoplasm of motor neurons in the spinal cord of G93A mutant SOD1-overexpressing mice in presymptomatic, onset, and postsymptomatic stages, and were, in particular, more intense in vacuolated neurons, than those in age-matched control mice. RT-qPCR analysis of MCP-1 mRNA disclosed age-related increases in G93A mice but not SJL mice, and significant increases in young to old G93A mice relative to the age-matched SJL mice. These observations are consistent with basic cell biological studies indicating the production of MCP-1 in developing human neurons and the NT2N human neuronal cell line [35,36]. Consistent with our findings, Henkel et al. reported increased levels of MCP-1 mRNA and protein in motor neurons as well as reactive glial cells in all stages of SOD1-mutated transgenic mouse models of ALS [20]. Another study demonstrated increased expression of MCP-1 in G93A mutant SOD1-expressing microglia [37,38]. These observations indicate that MCP-1 could be produced by motor neurons and glial cells in the spinal cord of SOD1-mutated ALS mice. However, it should be considered with the caveat that the discrepancy of staining intensity of MCP-1 in glial cells between the present and previous studies may result from differences in the methodologies used.

### Morphological and quantitative evaluations for CCR2 in SOD1-mutated mice

It is known that CCR2 acts as a membrane-bound receptor for the specific ligand MCP-1. CCR2 expression is regulated at a low level under physiological conditions [39], whereas it is upregulated by inflammatory stimuli [40]. In several tissues other than the CNS, CCR2 is constitutively expressed in monocytes and macrophages on their cell surface. In the CNS, it has been shown that CCR2 is expressed in microglia and is upregulated under pathological conditions such as multiple sclerosis, Alzheimer’s disease, and traumatic brain injury [30,41,42]. In the present study, the double-labeled immunofluorescence staining method revealed that CCR2 immunoreactivity was intense and exclusively localized in reactive astrocytes in the spinal cord of G93A mice at onset and postsymptomatic stages but not SJL mice at any stage. Several studies have provided evidence that astrocytes express CCR2 as the following: (1) MCP-1 and CCR2 are colocalized in astrocytes but not microglia in rat models of experimental autoimmune encephalomyelitis [43]; (2) MCP-1-driven astrocytic activation is associated with CCR2 induction mediated through activation of Akt and NF-κB [44]; (3) primary cultures derived from human and simian astrocytes express CCR2 mRNA and upregulate CCR2 by stimulation of TNFα and IFNγ [40]; (4) cultured human astrocytes express CCR2 mRNA and protein and perform chemotaxis and calcium influx in response to MCP-1 stimuli [45]. These observations support our data and suggest that CCR2-expressing astrocytes survive and demonstrate astrocytosis occurring in the advanced stage of a mutant SOD1 transgenic mouse of ALS.

Under physiological conditions, astrocytes behave as architectural components as well as participate in neuroprotective mechanisms, forming morphological and functional bases of the CNS. On the other hand, astrocytes are involved in several pathological conditions by exerting diverse effects on lesional microenvironments [46]. In particular, astrocytes are implicated in the pathomechanisms of neurological disorders, including Alzheimer’s disease [47], Parkinson’s disease [48], ALS [49,50], multiple sclerosis [51], and cerebral ischemia [52] via inflammatory responses. Relevantly, recent evidence that selective excision of a mutated SOD1 gene in astrocytes inhibited microglial activation and slowed disease progression suggests that mutant SOD1-expressing astrocytes are responsible for non-cell autonomous motor neuron death mediated through inflammatory mechanisms on the basis of crosstalk to microglia [53].

In the present study, we investigated CCR2 mRNA and protein expression levels in the spinal cord of SJL and G93A mice. In SJL mice, both the mRNA and protein levels were constantly low at presymptomatic, onset, and postsymptomatic stages. In G93A mice, CCR2 mRNA levels were increased in presymptomatic and onset stages but decreased in postsymptomatic stage, whereas CCR2 protein levels were significantly higher in the postsymptomatic G93A group than the age-matched SJL group. The discrepancy in expression levels between CCR2 mRNA and protein in postsymptomatic G93A mice may reflect certain mechanisms based on SOD1 mutation. It has been shown that over 30% of genes exhibit significantly divergent patterns of mRNA and protein levels in *Streptomyces coelicolor* and that the mRNA-protein discordance is attributable to differences in protein translation and degradation rates [54]. The stability of CCR2 protein in G93A mice might be changed by proteasome inhibition, which may occur in the presence of oxidative stress originating in mutant SOD1 toxicity [55]. CCR2 mRNA levels in human monocytes are also downregulated by treatment with bacteria-derived toxins such as lipopolysaccharide [56]. In cultured human monocytes, mRNA expression levels of the major chemokine receptors, CCR2, CCR5, and CXCR4 are upregulated by treatment with reactive oxygen species, including hydrogen peroxide, and are downregulated by treatment with antioxidant reagents such as pyrrolidine dithiocarbamate and *N*-acetylcysteine, although these treatments do not influence the stability of CCR2 protein on the cell surface [57]. Irradiation-triggered oxidative stress induces CCR2 protein expression associated with the lipid peroxidation product 4-hydroxy-2-nonenal in mouse hippocampi [58]. Moreover, a recent study indicated reduced CCR2 mRNA levels in circulating monocytes from sporadic ALS patients [22]. These observations suggest that altered redox states in G93A mice contribute to downregulation of CCR2 mRNA and upregulation or stabilization of CCR2 protein, leading to an increased innate immune response to SOD1 mutation-related oxidative stress.

### MCP-1 induces proliferation of astrocytes derived from SOD1-mutated mice

It is known that neuroinflammation based on activation of astrocytes and microglia diminishes survival of motor neurons to exacerbate disease progression of ALS [4]. Accumulating evidence suggests that astrocytes expressing mutant SOD1 are highly toxic to motor neurons. In particular, recent studies indicated that cultured astrocytes expressing mutant SOD1 demonstrated increased proliferation activity and reduced glutamate transporter-1 expression. The mutant SOD1-expressing astrocytes seems to produce certain soluble factors, which are toxic to motor neurons and activate microglia to induce motor neuron death [50,59]. In the present study, the basic and MCP-1-driven levels of proliferation activity and CCR2 expression were significantly increased in cultured astrocytes derived from G93A mice as compared to those from SJL mice. Moreover, the MCP-1-driven proliferation activity in the G93A astrocytes was suppressed by a CCR2 antagonist. Given the age-related increase in MCP-1 mRNA levels in the spinal cord of G93A mice, it is evident that astrocytes carrying a transgene for mutant SOD1 play a pivotal role in the disease progression via MCP-1/CCR2-mediated signaling.

## Conclusions

Taken together, we here showed a significant upregulation of MCP-1 and CCR2 in the spinal cord of G93A mutant human SOD1-overexpressing mice relative to nontransgenic littermates. This upregulation occurred even if in presymptomatic stage and was then enhanced along with aging. While MCP-1 was mainly expressed in motor neurons, CCR2 was mainly expressed in reactive astrocytes. These results provide in vivo evidence that MCP-1, released from the lesional cells including motor neurons, selectively stimulates CCR2-expressing astrocytes in a paracrine manner, leading to cell activation such as proliferation. Our results suggest that astrocytic activation driven by the MCP-1/CCR2 signaling pathway is a newly identified target of ALS therapies. Finally, determining the precise role of the MCP-1/CCR2 signaling pathway in SOD1-mutated human ALS requires further investigations.

## Methods

### Animals

The present study was approved by the Animal Research Ethics Committee of Tokyo Women’s Medical University. Mice overexpressing a transgene for G93A mutant human SOD1 [high expresser G1H line (G1H+/−) mice] [60] and nontransgenic littermates [background strain of Jackson Laboratory line (SJL) mice] were obtained from Jackson Laboratory (Bar Harbor, ME, USA). We maintained G1H+/− mice by mating transgenic males with nontransgenic females. Transgenic offsprings were genotyped by detecting human SOD1 protein in addition to mouse SOD1 protein using immunoblots as described before [5], and nontransgenic littermates were used as negative controls of the genetic background. After birth, G1H+/− mice appeared clinically intact at 9 w (presymptomatic stage), began to show clinical symptoms such as weakness and tremor prominent in their hindlimbs at around 12 w (onset stage), developed progressive gait disturbance reminiscent of human ALS (postsymptomatic stage), and died of respiratory failure or an eating disability by 20 w. Both G1H+/− and SJL mice were divided into the presymptomatic, onset, and postsymptomatic groups, and were sacrificed under anesthesia with ether ethanol at the respective periods (9 w, 12 w, and 15 w) to obtain lumbar spinal cords, including the main lesions in the mouse ALS-like disease.

### RT-qPCR analysis

The primer sets used in RT-qPCR are summarized in Table 1. All of them were purchased from Takara. Total RNA was extracted from freshly frozen materials of lumbar spinal cords using the RNeasy Lipid Tissue Mini kit (Qiagen, Valencia, CA, USA), and in turn were used for RT to obtain cDNA using the Prime Script RT-PCR kit (Takara, Tokyo, Japan). qPCR was performed using cDNA derived from 50 ng of total RNA, primer sets at a final concentration of 50 pM, and SYBR Premix Ex Taq II (Takara) according to the manufacturer’s instructions. Amplification profiles consisted of 95°C for 10 sec (initial denaturing), followed by 45 cycles at 95°C for 5 sec (denaturing), 55°C for 10 sec (annealing) and 72°C for 20 sec (extension). At the end of each run, a melting point analysis was performed to validate the specificity of the PCR products. The quality of the PCR products was also confirmed by ethidium bromide-supplemented agarose gel electrophoresis. House-keeping gene for glyceraldehyde dehydrogenase (GAPDH) was used to normalize transcription levels of MCP-1 and CCR2. The normalized data were compared between the different groups (n = 6 in each group).

**Table 1:** Primer sets for reverse transcription-quantitative polymerase chain reaction

**Gene**	**Sequence**	**Amplicon (bp)**
MCP-1	F: 5′-GCATCCACGTGTTGGCTCA-3′	95
	R: 5′-CTCCAGCCTACTCATTGGGATCA-3′	
CCR2	F: 5′-ACAGCTCAGGATTAACAGGGACTTG-3′	129
	R: 5′-ACCACTTGCATGCACACATGAC-3′	
GAPDH	F: 5′-TGTGTCCGTCGTGGATCTGA-3′	150
	R: 5′-TTGCTGTTGAAGTCGCAGGAG-3′	

Abbreviations: *MCP-1*, monocyte chemoattractant protein-1; *CCR2*, CC chemokine receptor 2; *GAPDH*, glyceraldehyde 3-phosphate dehydrogenase; *F*, forward; *R*, reverse; *bp*, base pair.

### Immunohistochemical analysis

The primary antibodies employed in immunohistochemistry are summarized in Table 2. NeuN and glial fibrillary acidic protein (GFAP) were used as markers for neurons and astrocytes, respectively. Both CD11b and Iba1 were used as markers for microglia. For immunohistochemistry, mice were perfused with phosphate-buffered saline, pH 7.5 (PBS) followed by 3% paraformaldehyde in PBS. Spinal cords were subsequently removed and processed for making paraffin-embedded materials or optimal cutting temperature compound-embedded frozen materials. Multiple 7-μm-thick paraffin-embedded sections and 10-μm-thick frozen sections were used for immunohistochemical staining. Paraffin-embedded sections were deparaffinized, and frozen sections were air-dried. These sections were subsequently rehydrated, quenched for 20 min in 3% hydrogen peroxide in PBS, pretreated for 30 min at room temperature with 3% bovine serum albumin in PBS, and in turn incubated overnight at 4°C with a primary antibody in PBS containing 0.1% Triton X-100 and 1% of normal horse serum. Antibody binding was visualized by the avidin-biotin-immunoperoxidase complex (ABC) method using the appropriate Vectastain ABC kit (Vector Laboratories, Burlingame, CA, USA) according to the manufacturer’s instructions. 3,3’-Diaminobenzidine tetrahydrochloride was the chromogen, and hematoxylin, the counterstain.

**Table 2:** Primary antibodies used for immunohistochemistry

**Antigen**	**Species**	**Dilution**	**Cat. No.**	**Source**
MCP-1	Rabbit	1:100	ab7202	Abcam
CCR2	Goat	1:100	sc-6228	SCB
CCR2	Goat	1:100	PA1-27409	Thermo
NeuN	Mouse	1:300	MAB377	Chemicon
GFAP	Rabbit	1:500	z0334	Dako
CD11b	Rat	1:50	ab8878	Abcam
Iba1	Rabbit	1:200	019-19741	Wako

Abbreviations: *MCP-1*, monocyte chemoattractant protein-1; *CCR2*, CC chemokine receptor 2; *GFAP*, glial fibrillary acidic protein; *Iba1*, ionized calcium-binding adaptor molecule 1; *SCB*, Santa Cruz Biotechnology.

Tissue distribution of MCP-1 and CCR2 was roughly verified by comparison with consecutive sections stained with hematoxylin-eosin (H&E). Immunohistochemical localization of CCR2 was precisely identified by the double-labeled immunofluorescence method. In brief, sections were incubated simultaneously with the primary antibodies against a target substance and a cell marker followed by the secondary antibodies such as Cy3-conjugated donkey anti-goat IgG and fluorescein isothiocyanate (FITC)-conjugated donkey anti-mouse, rat, or rabbit IgG (each diluted 1:200; Jackson Immunoresearch Laboratory, West Grove, PA, USA). DAPI was use as a nuclear stain. Immunoreaction product deposits were observed and recorded with a fluorescence microscope (Nikon ECLIPSE TS100; Nikon, Tokyo, Japan) or a confocal laser microscope (LSM 510 Meta, Carl Zeiss, Jena, Germany). The percentage of CCR2-immunoreactive cells in neurons, astrocytes, and microglia in the ventral horns was verified by NIH image J software.

### Immunoblot analysis

Resected fresh mouse spinal cords were stored at −80°C until use. For immunoblotting, frozen spinal cord materials were homogenized in 20 mM Tris-buffered saline, pH 8.5 (TBS), supplemented with 5 mM ethylenediaminetetraacetic acid (EDTA), 10% glycerol, 1% Triton X-100, 0.1% sodium dodecyl sulfate (SDS), 0.5% sodium deoxycholic acid, 1 mM phenylmethylsulfonylfluoride, and a protease inhibitor cocktail Complete Mini (Roche Diagnostics, Mannheim, Germany) according to the manufacturer’s instructions. The homogenate was then centrifuged at 12,500 *g* for 15 min to obtain supernatant containing total protein extracts. Protein concentration was determined by the Bradford method [61]. Total protein extracts were boiled for 10 min at 100°C with an equal volume of Laemli’s buffer containing 0.05% bromophenol blue, and were used for 12% sodium dodecyl sulfate-polyacrylamide gel electrophoresis. Aliquots of samples (70 μg of protein per lane) were loaded and separated in a gel, were and electroblotted onto a polyvinylidene difluoride (PVDF) membrane (Millipore, Billerica, MA, USA). After transfer, PVDF membranes were pretreated overnight at 4°C in 100 mM TBS, containing 0.1% Tween-20 and 5% skim milk, and then incubated for 1 h at room temperature with the anti-CCR2 antibody (Santa Cruz) at a dilution of 1:1,000 or mouse anti-β-actin antibody (Sigma-Aldrich, St. Louis, MO, USA) at a dilution of 1:2,000. Blots processed with omission of the primary antibodies served as negative reaction controls. Immunoreactive signals were visualized by the chemiluminescence method using the appropriate ECL detection system kit (Amersham, Buckinghamshire, UK), scanned with a Light-Capture Cooled Camera system (ATTO, Tokyo, Japan), and imported onto a personal computer. Optical density was then quantified with NIH Image J software. In each sample, immunoreactive signals for CCR2 were normalized by those for β-actin, and the CCR2/β-actin optical density ratio was compared between the different groups.

### Cell culture and proliferation assay

Astrocytes were grown in primary culture as described previously [62]. Briefly, the cerebral hemispheres of newborn SJL and G1H+/− mice were removed, the meninges were carefully removed off, and the cerebral tissues were dissociated with trypsin. The dissociated cells were seeded at 1.4 × 10^4^ viable cells/cm^2^ in a plastic culture flask and grown for 2 weeks in Dulbecco’s modified Eagle’s medium with 20% F-12 and 10% fetal bovine serum at 37°C in a 5%CO_2_ incubator. Before experiments, immunocytochemistry confirmed that over 95% of the cells were stained positively for the astrocytic marker GFAP. For proliferation assay, cells were plated on 96-well plates (4 × 10^3^ cells/well) and allowed to adhere for 24 h at 37°C. The cultures were then stimulated with recombinant murine MCP-1 (rmMCP-1; Pepro Tech, Rocky Hill, NJ, USA) at concentrations of 0, 1, 10 and 50 ng/mL for 48 h in the presence or absence of a CCR2 antagonist (Calbiochem, La Jolla, CA, USA) at a final concentration of 10 μM, followed by incubation with a Cell Counting Kit-8 (CCK-8; Dojindo Laboratories, Kumamoto, Japan) solution at a final concentration of 10 μM, and the cells were incubated for 2 h at 37°C, according to the manufacturer’s instructions. The optical absorbance at 450 nm for each sample was measured using a microplate reader (Bio-Lad Laboratories, Richmond, CA, USA).

### Statistics

Data were compared between three or more groups by two-way analysis of variance (ANOVA) followed by posthoc Bonferroni correction. Significance was considered in the case of P-value < 0.05.

## Competing interests

The authors declare that they have no competing interest.

## Author’s contributions

MKN performed most experiments. TY, YK and YI carried out in part the morphological and quantitative analyses. NS participated in the study design and coordination, and helped to draft the manuscript. All authors read and approved the final manuscript.

## References

[B1] Ince PG Highley JR Kirby J Wharton SB Takahashi H Strong MJ Shaw PJ Molecular pathology and genetic advances in amyotrophic lateral sclerosis: an emerging molecular pathway and the significance of glial pathology Acta Neuropathol 2011 1 657 671 10.1007/s00401-011-0913-0 22105541

[B2] Rowland LP Shneider NA Amyotrophic lateral sclerosis N Engl J Med 2001 1 1688 1700 10.1056/NEJM200105313442207 11386269

[B3] Gurney ME Pu H Chiu AY Dal Canto MC Polchow CY Alexander DD Caliendo J Hentati A Kwon Y Deng HX Motor neuron degeneration in mice that express a human Cu,Zn superoxide dismutase mutation Science 1994 1 1772 1775 10.1126/science.8209258 8209258

[B4] McCombe PA Henderson RD The Role of immune and inflammatory mechanisms in ALS Curr Mol Med 2011 1 246 254 10.2174/156652411795243450 21375489 PMC3182412

[B5] Shibata N Kawaguchi-Niida M Yamamoto T Toi S Hirano A Kobayashi M Effects of the PPARγ activator pioglitazone on p38 MAP kinase and IκBα in the spinal cord of a transgenic mouse model of amyotrophic lateral sclerosis Neuropathology 2008 1 387 398 10.1111/j.1440-1789.2008.00890.x 18312546

[B6] Conductier G Blondeau N Guyon A Nahon JL Rovère C The role of monocyte chemoattractant protein MCP1/CCL2 in neuroinflammatory diseases J Neuroimmunol 2010 1 93 100 10.1016/j.jneuroim.2010.05.010 20681057

[B7] Gu L Tseng SC Rollins BJ Monocyte chemoattractant protein-1 Chem Immunol 1999 1 7 29 10550927 10.1159/000058723

[B8] Li MQ Li HP Meng YH Wang XQ Zhu XY Mei J Li DJ Chemokine CCL2 enhances survival and invasiveness of endometrial stromal cells in an autocrine manner by activating Akt and MAPK/Erk1/2 signal pathway Fertil Steril 2012 1 4 919 929 10.1016/j.fertnstert.2011.12.049 22265030

[B9] Loberg RD Day LL Harwood J Ying C St John LN Giles R Neeley CK Pienta KJ CCL2 is a potent regulator of prostate cancer cell migration and proliferation Neoplasia 2006 1 578 586 10.1593/neo.06280 16867220 PMC1601934

[B10] Lu Y Cai Z Galson DL Xiao G Liu Y George DE Melhem MF Yao Z Zhang J Monocyte chemotactic protein-1 (MCP-1) acts as a paracrine and autocrine factor for prostate cancer growth and invasion Prostate 2006 1 1311 1318 10.1002/pros.20464 16705739

[B11] El Khoury J Luster AD Mechanisms of microglia accumulation in Alzheimer's disease: therapeutic implications Trends Pharmacol Sci 2008 1 626 632 10.1016/j.tips.2008.08.004 18835047

[B12] Huang DR Wang J Kivisakk P Rollins BJ Ransohoff RM Absence of monocyte chemoattractant protein 1 in mice leads to decreased local macrophage recruitment and antigen-specific T helper cell type 1 immune response in experimental autoimmune encephalomyelitis J Exp Med 2001 1 713 726 10.1084/jem.193.6.713 11257138 PMC2193420

[B13] Hughes PM Allegrini PR Rudin M Perry VH Mir AK Wiessner C Monocyte chemoattractant protein-1 deficiency is protective in a murine stroke model J Cereb Blood Flow Metab 2002 1 308 317 11891436 10.1097/00004647-200203000-00008

[B14] Baron P Bussini S Cardin V Corbo M Conti G Galimberti D Scarpini E Bresolin N Wharton S Shaw PJ Silani V Production of monocyte chemoattractant protein-1 in amyotrophic lateral sclerosis Muscle Nerve 2005 1 541 544 10.1002/mus.20376 15962273

[B15] Gupta PK Prabhakar S Sharma S Anand A A predictive model for amyotrophic lateral sclerosis (ALS) diagnosis J Neurol Sci 2012 1 68 72 10.1016/j.jns.2011.08.021 21907354

[B16] Henkel JS Engelhardt JI Siklós L Simpson EP Kim SH Pan T Goodman JC Siddique T Beers DR Appel SH Presence of dendritic cells, MCP-1, and activated microglia/macrophages in amyotrophic lateral sclerosis spinal cord tissue Ann Neurol 2004 1 221 235 10.1002/ana.10805 14755726

[B17] Simpson EP Henry YK Henkel JS Smith RG Appel SH Increased lipid peroxidation in sera of ALS patients: a potential biomarker of disease burden Neurology 2004 1 1758 1765 10.1212/WNL.62.10.1758 15159474

[B18] Wilms H Sievers J Dengler R Bufler J Deuschl G Lucius R Intrathecal synthesis of monocyte chemoattractant protein-1 (MCP-1) in amyotrophic lateral sclerosis: further evidence for microglial activation in neurodegeneration J Neuroimmunol 2003 1 139 142 10.1016/j.jneuroim.2003.08.042 14597108

[B19] Beers DR Zhao W Liao B Kano O Wang J Huang A Appel SH Henkel JS Neuroinflammation modulates distinct regional and temporal clinical responses in ALS mice Brain Behav Immun 2011 1 1025 1035 10.1016/j.bbi.2010.12.008 21176785 PMC3096756

[B20] Henkel JS Beers DR Siklós L Appel SH The chemokine MCP-1 and the dendritic and myeloid cells it attracts are increased in the mSOD1 mouse model of ALS Mol Cell Neurosci 2006 1 427 437 10.1016/j.mcn.2005.10.016 16337133

[B21] Mantovani S Garbelli S Pasini A Alimonti D Perotti C Melazzini M Immune system alterations in sporadic amyotrophic lateral sclerosis patients suggest an ongoing neuroinflammatory process J Neuroimmunol 2009 1 73 79 10.1016/j.jneuroim.2009.02.012 19307024

[B22] Zhang R Gascon R Miller RG Gelinas DF Mass J Lancero M Narvaez A McGrath MS MCP-1 chemokine receptor CCR2 is decreased on circulating monocytes in sporadic amyotrophic lateral sclerosis (sALS) J Neuroimmunol 2006 1 87 93 10.1016/j.jneuroim.2006.06.008 16857270

[B23] Dawson J Miltz W Mir AK Wiessner C Targeting monocyte chemoattractant protein-1 signalling in disease Expert Opin Ther Targets 2003 1 35 48 10.1517/14728222.7.1.35 12556201

[B24] Roebuck KA Carpenter LR Lakshminarayanan V Page SM Moy JN Thomas LL Stimulus-specific regulation of chemokine expression involves differential activation of the redox-responsive transcription factors AP-1 and NF-κB J Leukoc Biol 1999 1 291 298 10080530 10.1002/jlb.65.3.291

[B25] Allavena P Bianchi G Zhou D van Damme J Jílek P Sozzani S Mantovani A Induction of natural killer cell migration by monocyte chemotactic protein-1, -2 and −3 Eur J Immunol 1994 1 3233 3236 10.1002/eji.1830241249 7805752

[B26] Amantea D Nappi G Bernardi G Bagetta G Corasaniti MT Post-ischemic brain damage: pathophysiology and role of inflammatory mediators FEBS J 2009 1 13 26 10.1111/j.1742-4658.2008.06766.x 19087196

[B27] Hinojosa AE Garcia-Bueno B Leza JC Madrigal JL CCL2/MCP-1 modulation of microglial activation and proliferation J Neuroinflammation 2011 1 77 10.1186/1742-2094-8-77 21729288 PMC3146846

[B28] Lee EO Park HJ Kang JL Kim HS Chong YH Resveratrol reduces glutamate-mediated monocyte chemotactic protein-1 expression via inhibition of extracellular signal-regulated kinase 1/2 pathway in rat hippocampal slice cultures J Neurochem 2010 1 1477 1487 10.1111/j.1471-4159.2009.06564.x 20050970

[B29] Mahad D Callahan MK Williams KA Ubogu EE Kivisäkk P Tucky B Modulating CCR2 and CCL2 at the blood–brain barrier: relevance for multiple sclerosis pathogenesis Brain 2006 1 212 223 16230319 10.1093/brain/awh655

[B30] Semple BD Bye N Rancan M Ziebell JM Morganti-Kossmann MC Role of CCL2 (MCP-1) in traumatic brain injury (TBI): evidence from severe TBI patients and CCL2−/− mice J Cereb Blood Flow Metab 2010 1 769 782 10.1038/jcbfm.2009.262 20029451 PMC2949175

[B31] Tanabe S Heesen M Berman MA Fischer MB Yoshizawa I Luo Y Dorf ME Murine astrocytes express a functional chemokine receptor J Neurosci 1997 1 6522 6528 9254664 10.1523/JNEUROSCI.17-17-06522.1997 PMC6573137

[B32] Kuhle J Lindberg RL Regeniter A Mehling M Steck AJ Kappos L Czaplinski A Increased levels of inflammatory chemokines in amyotrophic lateral sclerosis Eur J Neurol 2009 1 771 774 10.1111/j.1468-1331.2009.02560.x 19236470

[B33] Philips T Robberecht W Neuroinflammation in amyotrophic lateral sclerosis: role of glial activation in motor neuron disease Lancet Neurol 2011 1 253 263 10.1016/S1474-4422(11)70015-1 21349440

[B34] Tateishi T Yamasaki R Tanaka M Matsushita T Kikuchi H Isobe N Ohyagi Y Kira J CSF chemokine alterations related to the clinical course of amyotrophic lateral sclerosis J uroimmunol 2010 1 76 81 10.1016/j.jneuroim.2010.03.004 20381883

[B35] Coughlan CM McManus CM Sharron M Gao Z Murphy D Jaffer S Expression of multiple functional chemokine receptors and monocyte chemoattractant protein-1 in human neurons Neuroscience 2000 1 591 600 10.1016/S0306-4522(00)00024-5 10828541

[B36] Meng SZ Oka A Takashima S Developmental expressison of monocyte chemoattractant protein-1 in the human cerebellum and brainstem Brain Dev 1999 1 30 35 10.1016/S0387-7604(98)00065-5 10082250

[B37] Butovsky O Siddiqui S Gabriely G Lanser AJ Dake B Murugaiyanf G Modulating inflammatory monocytes with a unique microRNA gene signature ameliorates murine ALS J Clin Invest 2012 1 3063 3087 10.1172/JCI62636 22863620 PMC3428086

[B38] Sargsyan SA Blackburn DJ Barber SC Monk PN Shaw PJ Mutant SOD1 G93A microglia have an inflammatory phenotype and elevated production of MCP-1 NeuroReport 2009 1 1450 1455 10.1097/WNR.0b013e328331e8fa 19752764 PMC2889291

[B39] Semple BD Kossmann T Morganti-Kossmann MC Role of chemokines in CNS health and pathology: a focus on the CCL2/CCR2 and CXCL8/CXCR2 networks J Cereb Blood Flow Metab 2010 1 459 473 10.1038/jcbfm.2009.240 19904283 PMC2949152

[B40] Croitoru-Lamoury J Guillemin GJ Boussin FD Mognetti B Gigout LI Chéret A Vaslin B Le Grand R Brew BJ Dormont D Expression of chemokines and their receptors in human and simian astrocytes: evidence for a central role of TNFα and IFNγ in CXCR4 and CCR5 modulation Glia 2003 1 354 370 10.1002/glia.10181 12555203

[B41] Hickman SE El Khoury J Mechanisms of mononuclear phagocyte recruitment in Alzheimer's disease CNS Neurol Disord Drug Targets 2010 1 168 173 10.2174/187152710791011982 20205643 PMC3684802

[B42] Tanuma N Sakuma H Sasaki A Matsumoto Y Chemokine expression by astrocytes plays a role in microglia/macrophage activation and subsequent neurodegeneration in secondary progressive multiple sclerosis Acta Neuropathol 2006 1 195 204 10.1007/s00401-006-0083-7 16733654

[B43] Jee Y Yoon WK Okura Y Tanuma N Matsumoto Y Upregulation of monocyte chemotactic protein-1 and CC chemokine receptor 2 in the central nervous system is closely associated with relapse of autoimmune encephalomyelitis in Lewis rats J Neuroimmunol 2002 1 49 57 10.1016/S0165-5728(02)00147-9 12098510

[B44] Quinones MP Kalkonde Y Estrada CA Jimenez F Ramirez R Mahimainathan L Role of astrocytes and chemokine systems in acute TNFalpha induced demyelinating syndrome: CCR2-dependent signals promote astrocyte activation and survival via NF-kappaB and Akt Mol Cell Neurosci 2008 1 96 109 10.1016/j.mcn.2007.08.017 17949991 PMC2894699

[B45] Andjelkovic AV Song L Dzenko KA Cong H Pachter JS Functional expression of CCR2 by human fetal astrocytes J Neurosci Res 2002 1 219 231 10.1002/jnr.10372 12271471

[B46] Sofroniew MV Vinters HV Astrocytes: biology and pathology Acta Neuropathol 2010 1 7 35 10.1007/s00401-009-0619-8 20012068 PMC2799634

[B47] Xia MQ Hyman BT Chemokines/chemokine receptors in the central nervous system and Alzheimer's disease J Neurovirol 1999 1 32 41 10.3109/13550289909029743 10190688

[B48] Rappold PM Tieu K Astrocytes and therapeutics for Parkinson's disease Neurotherapeutics 2010 1 413 423 10.1016/j.nurt.2010.07.001 20880505 PMC2948546

[B49] Ferraiuolo L Higginbottom A Heath PR Barber S Greenald D Kirby J Shaw PJ Dysregulation of astrocyte-motoneuron cross-talk in mutant superoxide dismutase 1-related amyotrophic lateral sclerosis Brain 2011 1 2627 2641 10.1093/brain/awr193 21908873 PMC3170534

[B50] Papadeas ST Kraig SE O'Banion C Lepore AC Maragakis NJ Astrocytes carrying the superoxide dismutase 1 (SOD1G93A) mutation induce wild-type motor neuron degeneration in vivo Proc Natl Acad Sci USA 2011 1 17803 17808 10.1073/pnas.1103141108 21969586 PMC3203804

[B51] van Der Voorn P Tekstra J Beelen RH Tensen CP Van Der Valk P De Groot CJ Expression of MCP-1 by reactive astrocytes in demyelinating multiple sclerosis lesions Am J Pathol 1999 1 45 51 10.1016/S0002-9440(10)65249-2 9916917 PMC1853444

[B52] Takano T Oberheim N Cotrina ML Nedergaard M Astrocytes and ischemic injury Stroke 2009 1 S8 12 10.1161/STROKEAHA.108.533166 19064795 PMC2653262

[B53] Yamanaka K Chun SJ Boillee S Fujimori-Tonou N Yamashita H Gutmann DH Takahashi R Misawa H Cleaveland DW Astrocytes as determinants of disease progression in inherited amyotrophic lateral sclerosis Nat Neurosci 2008 1 251 253 10.1038/nn2047 18246065 PMC3137510

[B54] Jayapal KP Philp RJ Kok YJ Yap MG Sherman DH Griffin TJ Hu WS Uncovering genes with divergent mRNA-protein dynamics in Streptomyces coelicolor PLoS One 2008 1 e2097 10.1371/journal.pone.0002097 18461186 PMC2367054

[B55] Kabashi E Durham HD Failure of protein quality control in amyotrophic lateral sclerosis Biochim Biophys Acta 2006 1 1038 1050 10.1016/j.bbadis.2006.06.006 16876390

[B56] Sica A Saccani A Borsatti A Power CA Wells TN Luini W Polentarutti N Sozzani S Mantovani A Bacterial lipopolysaccharide rapidly inhibits expression of C-C chemokine receptors in human monocytes J Exp Med 1997 1 969 974 10.1084/jem.185.5.969 9120403 PMC2196159

[B57] Saccani A Saccani S Orlando S Sironi M Bernasconi S Ghezzi P Mantovani A Sica A Redox regulation of chemokine receptor expression Proc Natl Acad Sci USA 2000 1 2761 2766 10.1073/pnas.97.6.2761 10716998 PMC16003

[B58] Limoli CL Giedzinski E Baure J Rola R Fike JR Redox changes induced in hippocampal precursor cells by heavy ion irradiation Radiat Environ Biophys 2007 1 167 172 10.1007/s00411-006-0077-9 17103219

[B59] Díaz-Amarilla P Olivera-Bravo S Trias E Cragnolini A Martínez-Palma L Cassina P Beckman J Barbeito L Phenotypically aberrant astrocytes that promote motoneuron damage in a model of inherited amyotrophic lateral sclerosis Proc Natl Acad Sci USA 2011 1 18126 18131 10.1073/pnas.1110689108 22010221 PMC3207668

[B60] Gurney ME Transgenic-mouse model of amyotrophic lateral sclerosis N Engl J Med 1994 1 1721 1722 10.1056/NEJM199412223312516 7832899

[B61] Sapan CV Lundblad RL Price NC Colorimetric protein assay techniques Biotechnol Appl Biochem 1999 1 99 108 10075906

[B62] Kawaguchi M Shibata N Horiuchi S Kobayashi M Glyoxal inactivates glutamate transporter-1 in cultured rat astrocytes Neuropathology 2005 1 27 36 10.1111/j.1440-1789.2004.00579.x 15822816

